# Extracellular Vesicles from Infected Cells Are Released Prior to Virion Release

**DOI:** 10.3390/cells10040781

**Published:** 2021-04-01

**Authors:** Yuriy Kim, Gifty A. Mensah, Sarah Al Sharif, Daniel O. Pinto, Heather Branscome, Sowmya V. Yelamanchili, Maria Cowen, James Erickson, Pooja Khatkar, Renaud Mahieux, Fatah Kashanchi

**Affiliations:** 1 Laboratory of Molecular Virology, School of Systems Biology, George Mason University, Manassas, VA 20110, USA; ykim78@gmu.edu (Y.K.); gmensah2@gmu.edu (G.A.M.); salshar@gmu.edu (S.A.S.); dpinto1@gmu.edu (D.O.P.); hbransco@gmu.edu (H.B.); mcowen4@gmu.edu (M.C.); jericks@gmu.edu (J.E.); pkhatkar@gmu.edu (P.K.); 2 Department of Anesthesiology, University of Nebraska Medical Center, Omaha, NE 68198, USA; syelamanchili@unmc.edu; 3 International Center for Research in Infectiology, Retroviral Oncogenesis Laboratory, INSERM U1111-Université Claude Bernard Lyon 1, Ecole Normale Superieure de Lyon, Université de Lyon, Fondation Pour La Recherche Médicale, Labex Ecofect, 69007 Lyon, France; renaud.mahieux@ens-lyon.fr

**Keywords:** HIV-1, HTLV-1, extracellular vesicles, exosomes, cART

## Abstract

Here, we have attempted to address the timing of EV and virion release from virally infected cells. Uninfected (CEM), HIV-1-infected (J1.1), and human T cell leukemia virus-1 (HTLV-1)-infected (HUT102) cells were synchronized in G_0_. Viral latency was reversed by increasing gene expression with the addition of serum-rich media and inducers. Supernatants and cell pellets were collected post-induction at different timepoints and assayed for extracellular vesicle (EV) and autophagy markers; and for viral proteins and RNAs. Tetraspanins and autophagy-related proteins were found to be differentially secreted in HIV-1- and HTLV-1-infected cells when compared with uninfected controls. HIV-1 proteins were present at 6 h and their production increased up to 24 h. HTLV-1 proteins peaked at 6 h and plateaued. HIV-1 and HTLV-1 RNA production correlated with viral protein expression. Nanoparticle tracking analysis (NTA) showed increase of EV concentration over time in both uninfected and infected samples. Finally, the HIV-1 supernatant from the 6-h samples was found not to be infectious; however, the virus from the 24-h samples was successfully rescued and infectious. Overall, our data indicate that EV release may occur prior to viral release from infected cells, thereby implicating a potentially significant effect of EVs on uninfected recipient cells prior to subsequent viral infection and spread.

## 1. Introduction

Since its discovery in 1981, human immunodeficiency virus type 1 (HIV-1), the causative agent of acquired immunodeficiency syndrome (AIDS), has caused approximately 32.7 million deaths worldwide [1]. In 2018 alone, there were 1.7 million new HIV-1 infections among adult and children [1]. Currently, there are no definitive cures for HIV-1/AIDS; however, the development of combination antiretroviral therapy (cART) has significantly improved the quality of life of HIV-1 patients and has kept the occurrence of HIV-1-related opportunistic infections at bay [2]. An estimated 24 million HIV-1 patients are presently receiving cART [1]. Antiretroviral therapy cocktails comprise drugs that target various stages of the HIV-1 life cycle including viral fusion, reverse transcription, integration, viral polyprotein cleavage and virion maturation [3]. Although cART does not completely eradicate the virus, it suppresses it by lowering the viral load to undetectable levels in plasma and requires a lifelong strict regimen [2]. Therefore, failure of medication adherence or discontinuation of cART leads to the reactivation of the virus [4]. Administration of cART forces infected cells into latency. Latency is one of the major hurdles when it comes to eliminating the virus in HIV-1 [5]. HIV-1 establishes viral reservoirs and persists in infected cells via integration of the provirus into the genome. Recently, it has been shown that latent reservoirs, particularly in resting memory CD4^+^ T cells and macrophages, are not completely silent as patients under cART have been shown to produce low levels of viral RNA in CD4^+^ T cells and in various regions of the brain [6,7]. Small membrane-bound particles known as extracellular vesicles (EVs) have been shown to contribute to this viral persistence.

EVs are membrane-contained organelles secreted by all cell types and play a major role as signal messengers in intercellular communication, making them crucial for multiple physiological and pathological processes [8,9]. Secreted EVs include a wide range of particles that can be classified based on their size and biogenesis pathway, such as exomeres (39–71 nm) [10], exosomes (50–100 nm) and microvesicles (100–1000 nm) [11]. EVs carry functional cargoes including viral proteins, lipids, cytokines, RNA and, in some cases, the virion itself (i.e., hepatitis B) [12,13]. As such, EVs are able to modulate disease pathogenesis in several types of infections such as HIV-1, HTLV-1, Ebola virus, and Rift Valley fever by promoting cell-to-cell spread, immune evasion, persistent inflammation, and cell cycle regulation in recipient cells [14,15,16,17,18]. Recently, we found that EVs from HIV-1-infected cells transport TAR RNA to naïve cells, compromising their resistance to HIV-1 through downregulation of apoptosis by depleting CDK9 levels [19]. Moreover, TAR RNA was found to upregulate the levels of proinflammatory cytokines by activation of the NF-kB pathway [20]. Furthermore, we observed increased production of a novel RNA transcript, TAR-*gag*, which we later determined to play a role in regulating viral transcription in infected cells [16,21]. In the case of HTLV-1 infection, EVs were found to facilitate cell-to-cell transmission [14].

EVs such as exosomes and RNA viruses including HIV-1 are close in size and are released via the same endosomal sorting complex required for transport (ESCRT) pathway [22,23]. As such, many viruses are able to hijack the mechanism of EV release and uptake to progress through their life cycle and facilitate infection [15,22]. Due to its dependence on the host machinery for its replication and release, HIV-1 manipulates cell cycle regulatory mechanisms to both promote cell cycle progression and cause its arrest [24,25]. Between mitosis and interphase, a cell, because of being affected by multiple anti-mitogenic factors, can enter a quiescent state called gap 0 phase (G_0_) that is characterized by division arrest [26]. HIV-1 infection results in the arrest or delay in the G_2_ phase in human T cells [24]. This process is mediated by HIV-1 accessory proteins Vpr and Vif, both of which have been detected in urinary EVs derived from HIV-1 patients [27]. Mechanistically, Vpr disturbs the cell cycle by preventing the activation of CDC2/CDK1, whereas Vif associates with BRD4 and CDK9 to speed up the G_1_/S transition [24,25]. CDK9, along with other proteins such as CDK2 and CDK4 central to the cell cycle, were found to be upregulated in T cell EVs [28]. Together, this signifies the potential crucial role EVs play in regulating the cell cycle.

HIV-1 packages and releases its viral products such as RNA in EVs to avoid detection by members of the host immune system [29]. To date, it is not clear whether there is a timing difference between EV and virion release from infected cells. In this current study, we aimed to understand whether EVs or virions are first released from the infected cell. Here, we demonstrated that EVs precede the secretion of viral particles. Data showed that both HIV-1 and HTLV-1-infected cells produce EVs with viral proteins and RNA before virion release beginning at 6 h. For HIV-1, this EV and virion release continued to increase until 24 h. A slightly different scenario was observed in HTLV-1 cells where EV and virion release peaked at 6 h but gradually declined afterwards. This timing could shed light on how the contents of EVs are able to increase the susceptibility of viral infection, perhaps by priming the environment prior to viral egress or altering the progression of the cell cycle. Overall, this study has the potential to contribute to the overall understanding of the role EVs play in viral infection and ways to inhibit viral spread.

## 2. Materials and Methods

### 2.1. Cell Culture and Treatment

CEM (uninfected T cells), J1.1 (HIV-1-infected T cells), and HUT102 (HTLV-1-infected T cells) cells were grown in complete RPMI 1640 media containing 1% L-glutamine, 1% streptomycin/penicillin and 10% exosome-free fetal bovine serum (FBS) at 37 °C and 5.0% CO_2_ for seven days. The cells were synchronized by serum starvation (1% FBS) for three days. The J1.1 cells were treated with cART drugs: 10 mM of indinavir (protease inhibitor) and 10 mM of emtricitabine (nucleoside reverse transcriptase inhibitor) for three days during serum starvation. The cells were then washed and plated in 20% FBS media prior to induction with phytohemagglutinin (PHA; 10 µg/µL) and IL-2. The samples were picked up at 0, 3, 6, 12, and 24 h post-induction. Peripheral blood mononuclear cells (PBMCs) were also grown in complete RPMI 1640 media at 37 °C and 5.0% CO_2_ for seven days; IL-2 and PHA were added daily. On the seventh day, the PBMCs were infected with the HIV-1 89.6 strain (multiplicity of infection (MOI):10.0) and treated again with PHA/IL-2 for three more days. On the third day post-infection, a cART cocktail was added. The infected PBMCs were then treated with cART and IL-7 to promote latency. On day 6 post-infection, the cells were serum-starved (1% FBS) for three days and then induced with 20% FBS media and PHA/IL-2. The samples were collected at 0, 6, and 24 h post-induction.

### 2.2. EV Isolation and Ultracentrifugation

The CEM, J1.1, and HUT102 cells were grown in complete RPMI media over a course of two weeks in T75 flasks, then synchronized by serum starvation as described previously. The J1.1 cells were treated with a cART drug cocktail during serum starvation. The cells were then washed and plated in 20% FBS media in two T25 flasks representing 6 and 24 h, respectively, and induced with PHA/IL-2. The cells were pelleted by centrifugation at 500× *g* for 10 min, and the cell supernatant was collected. An additional centrifugation at 2000× *g* for 10 min was used to pellet dead cells and cell debris. The supernatant was collected and ultracentrifuged in a Ti70 rotor (Beckman Coulter; Indianapolis, IN, USA). For total EVs, a 100,000× *g* spin was performed for 90 min to pellet all EVs. The pellets were then resuspended in 50 µL PBS. All centrifugations were performed at 4 °C.

### 2.3. Virus Rescue Assay

The J1.1 (HIV-1-infected T cells) and HUT102 (HTLV-1-infected T cells) supernatants from 6 and 24 h were used to treat naïve CEM, Jurkat, U937, and THP-1-derived dendritic cells ((TNF-α (20 ng/mL), ionomycin (200 ng/mL), IL-4 (10 ng/µL), and GM-CSF (100 U/µL)). A total of 10^6^ naïve cells were resuspended in 400 µL supernatant and incubated for two days. Afterwards, 600 µL fresh complete RPMI1640 medium was added and the cells were allowed to incubate for two days. The cells were then harvested and pelleted for Western blot analysis.

### 2.4. EV and Virion Capture with Nanotrap Particles

For EV and virion isolation from samples, we utilized Nanotrap particles (Ceres Nanosciences, Inc.; Manassas, VA, USA). Equal amounts of each Nanotrap particle (NT80, NT82, NT86) and 1X PBS without calcium and magnesium were mixed and resuspended to make a slurry. For the capture of EVs and virions from supernatants, 60 µL slurry was added to 1 mL supernatant and the samples were left rotating overnight at 4 °C. The particles were separated, washed with PBS, and the pellets were resuspended (50 µL PBS for RNA isolation or 20 µL Laemmli buffer for Western blotting) for downstream assays.

### 2.5. Preparation of Whole Cell Extracts and Western Blot Analysis

The cells were centrifuged and washed with PBS. The pellet was resuspended in a lysis buffer ((50 mM Tris-HCl (pH 7.5), 120 mM NaCl, 5 mM EDTA, 0.5% Nonidet P-40, 50 mM NaF, 0.2 mM Na3VO4, 1 mM dithiothreitol (DTT), and 1 complete protease inhibitor cocktail tablet per 50 mL (Roche Applied Science, Mannheim, Germany)). The mixture was incubated on ice for 25 min with vortexing every 5 min. Cell debris was separated via centrifugation at 12,000× *g* at 4 °C for 10 min. The protein concentration was measured using the Bradford assay according to the manufacturer’s guideline (Bio-Rad, Hercules, CA, USA).

For Western blot analysis, the samples were mixed with Laemmli buffer and heated. Fifteen to twenty microliters of each sample were loaded onto a 4–20% Tris/glycine 1.0 mm gel (Invitrogen, Carlsbad, CA, USA). The samples were run at 160 V for an hour and transferred onto PVDF membranes (Millipore, Burlington, MA, USA) at 50 mA overnight. The membranes were blocked in 5% milk in PBS-T (PBS with 0.1% Tween-20) for 30 min at 4 °C prior to an overnight incubation at 4 °C in PBS-T with the appropriate primary antibody against the proteins of interest. The next day, the membranes were washed and incubated with the appropriate HRP-conjugated secondary antibody for 2 h at 4 °C. HRP luminescence was activated with a Clarity Western enhanced chemiluminescence (ECL) Substrate (Bio-Rad, Hercules, CA, USA) and imaged using a ChemiDoc Touch system (Bio-Rad, Hercules, CA, USA).

### 2.6. RNA Isolation and RT-qPCR

For the quantitative characterization of HIV-1 RNA, total RNA was purified from cell pellets and EV/virion-enriched NT80/82/86 pellets. RNA was isolated using a Trizol Reagent (Invitrogen, Carlsbad, CA, USA) according to the manufacturer’s protocol. Total RNA was used to generate cDNA with a GoScript Reverse Transcription System (Promega, Madison, WI, USA) using specific reverse primers HIV-1 TAR Reverse (5′-CAA CAG ACG GGC ACA CAC TAC-3′, Tm = 58 °C), HIV-1 Gag Reverse (5′-GCT GGT AGG GCT ATA CAT TCT TAC-3′; Tm = 54 °C) and HIV-1 Envelope Reverse (5′-TGG GAT AAG GGT CTG AAA CG-3′; Tm = 58 °C). RT-qPCR was performed on the generated cDNA samples with the use of TAR Reverse (5′-CAA CAG ACG GGC ACA CAC TAC-3′, Tm = 58 °C) and TAR Forward (5′-GGT CTC TCT GGT TAG ACC AGA TCT G-3′, Tm = 60 °C) primers. DNA serial dilutions from the HIV-1-infected 8E5 cells were used as standards as described [30].

For the quantitative analysis of HTLV-1 RNA, after isolation as described above, cDNA was generated with a GoScript kit using an oligo(dT) reverse primer. RT-qPCR analysis was performed as described earlier [14] using specific primers: HTLV-1 *env* (env-Reverse 5′-CCA TCG TTA GCG CTT CCA GCC CC-3′, Tm = 64.4 °C; env-Forward 5′-CGG GAT CCT AGC GTG GGA ACA GGT-3′, Tm = 64.5°C), HTLV-1 *tax* (tax-Reverse 5′- AAC ACG TAG ACT GGG TAT CC-3′, Tm = 53.6 °C; tax-Forward 5′- ATC CCG TGG AGA CTC CTC AA-3′, Tm = 57.6 °C). DNA from the HUT102 cells was used as quantitative standards.

### 2.7. Nanoparticle Tracking Analysis

Quantification of EVs was performed using the ZetaView Z-NTA (Particle Metrix, Inning am Ammersee, Germany) and its corresponding software package. The machine was calibrated with the use of 100 nm polystyrene nanoparticles (Applied Microspheres, Leusden, Netherlands) prior to sample readings at a sensitivity of 65 and a minimum brightness of 20. For each measurement, the instrument pre-acquisition parameters were set to a temperature of 23 °C, a sensitivity of 85, 30 frames per second, and a shutter speed of 250. For each measurement, 1 mL of the sample diluted in deionized (DI) water was loaded into the cell, and the instrument measured each sample at 11 different positions throughout the cell, with three readings at each position. After automated analysis and removal of any outliers from the 11 positions was completed, the concentration, mean, median, and mode sizes of the samples were calculated using the ZetaView 8.04.02 software and analyzed using the same software and Microsoft Excel.

### 2.8. Statistical Analysis

Standard deviations were calculated for the quantitative experiments done in triplicate using Microsoft Excel. All p-values were calculated using a two-tailed student’s *t*-test and were considered to be statistically significant when *p*  <  0.05 (*), of great significance when *p*  <  0.01 (**), of greater significance when *p* < 0.001 (***), and of greatest significance when *p* < 0.0001 (****).

## 3. Results

### 3.1. Enrichment and Characterization of the EVs and Virions Released over Time from HIV-1-Infected Cells

It was previously shown that EVs from virally infected cells play a major role in the pathogenesis of viral infections, including contribution to inflammation, long-term chronic conditions such as HIV-1-associated neurocognitive disorder (HAND), immune cell dysfunction, and viral spread [16,17,19,20,31,32,33]. Additionally, EVs from virally infected cells contain viral RNA and proteins that play a role in disease pathogenesis through the bystander effect as they carry viral products that contribute to the pathogenicity of the infection. Here, we decided to investigate whether the release of EVs precedes the release of virions, both may be released at the same time or virions are released prior to EV release. Therefore, to better control release of either particle, we initially synchronized cells, since most cells in a cell culture are a disproportionate mixture of cells at G_1_, S or G_2_/M and more than 50% are at G_1_. We synchronized all the uninfected and infected cells at G_0_ followed by release using complete media containing 20% serum, IL-2 and PHA to reverse latency and induce HIV-1 transcription. The supernatant samples were collected at 0, 3, 6, 12, and 24 h post-induction timepoints and enriched for virus and EVs using NT80/82/86 nanoparticles and subsequently processed for downstream assays. Previously, we showed that these three nanoparticles are capable of binding to EVs and viruses [34].

**Figure 1:**
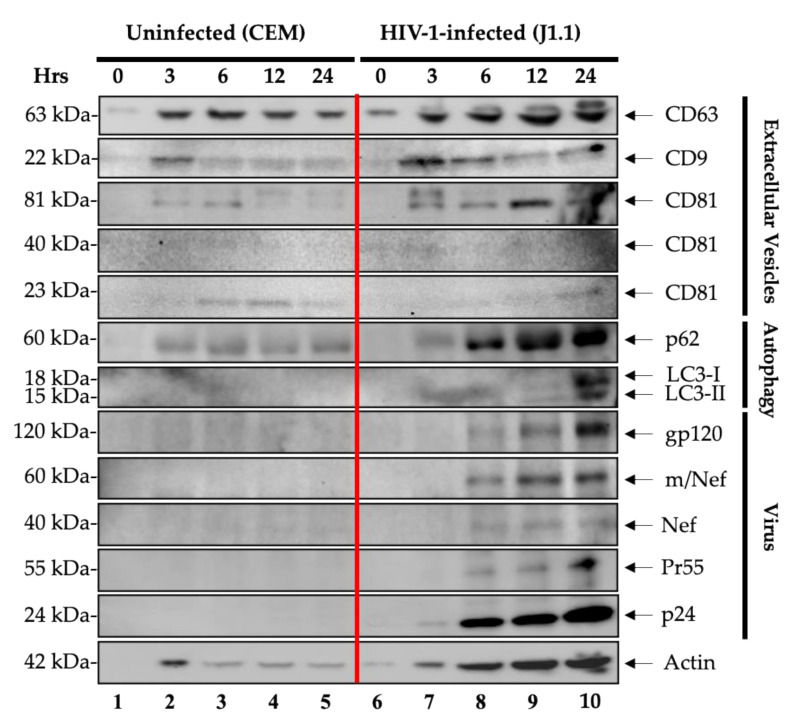
Enrichment and characterization of EVs and virions released over time from HIV-1-infected lymphocytes. CEM and J1.1 cells (5 × 10^7^ cells/mL) were serum-starved in 1% fetal bovine serum (FBS) media to induce cell cycle synchronization (G_0_ stage of the cell cycle) and incubated with cART. Refeeding with complete media containing 20% EV-depleted serum and PHA/IL-2 to increase HIV-1 transcription and reverse latency. The supernatants were collected at 0, 3, 6, 12, and 24 h post-induction and enriched for viral particles and EVs using Nanotrap particles (NT80/82/86) overnight at 4 °C. Nanotrapped pellets for the CEM (lanes 1–5) and J1.1 cells (lanes 6–10) were analyzed by Western blotting for exosomal markers (CD63, CD9, and CD81), autophagy markers (p62, LC3-I, and LC3-II), HIV-1 viral proteins (gp120, m/Nef, Nef, Pr55, and p24), and actin as a control.

The data in Figure 1 indicate that EV markers such as CD63 and CD9 were present at 3 h post-release and the levels of CD63 increased in the HIV-1-infected cells up to 24 h. Overall, there also was an increase of CD9 from the infected cells, as well as of the CD81 marker. These data collectively indicate that tetraspanin family members (i.e., CD63, CD9, and CD81) may be altered in expression and release from infected cells. We next focused on markers of autophagy including p62 and LC3-I/II family members. These markers are indicative of how autophagosomes are formed and whether they are released from infected cells. As expected, there was more p62 and LC3-I/II release from the HIV-1-infected cells starting at 6 h post-release. This is consistent with the notion that HIV-1-infected cells normally allow ample gene expression which may ultimately be regulated by the autophagy pathway. For instance, cells that have excess viral RNA or proteins made either have to digest the unwanted RNA/protein complex or have to package and release it due to blocking of the autophagosome maturation stemming from the presence of viral proteins such as Env, Tat, Nef or Vpr [35]. Finally, we looked for presence of viral proteins from the released cells and probed for presence of gp120, Nef, p24 and Pr55 (Gag precursor of p24). Interestingly, Western blot analysis showed presence of all proteins starting at 6 h and increased over time up to 24 h. Collectively, these data indicate that markers of EVs are present prior to viral markers and that they all increase over 24 h after G_0_ release.

### 3.2. Increased Levels of Intracellular and Extracellular Viral RNA Post-Release

**Figure 2:**
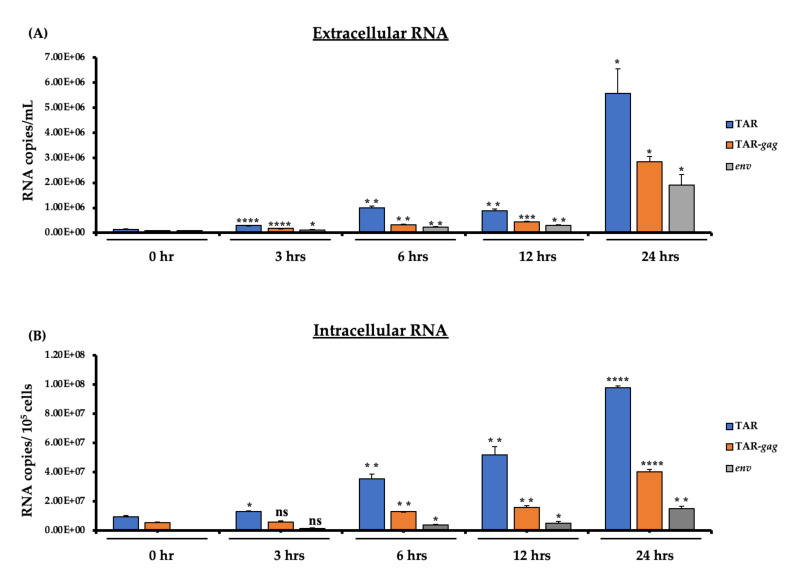
Levels of viral RNA content of secreted EVs and virions from HIV-1-infected cells. J1.1 cells (5 × 10^7^ cells/mL) were forced into G_0_ by growing in serum-starved 1% fetal bovine serum (FBS) media and were also treated with cART for three days. The cells were washed and cultured in complete media containing 20% EV-depleted serum and PHA/IL-2 to stimulate HIV-1 transcription and reverse latency. (**A**) The supernatants were collected at 0, 3, 6, 12, and 24 h and enriched for HIV-1 viral particles and EVs using NT80/82/86 via overnight incubation at 4 °C. (**B**) The cell pellets were harvested at 0, 3, 6, 12, and 24 h. RNA from nanotrapped pellets (extracellular; panel (**A**)) and cell pellets (intracellular; panel (**B**)) were extracted and analyzed by RT-qPCR for viral RNA production (TAR, TAR-*gag* and *env*). RT-qPCRs were performed in technical triplicate. Student’s *t*-tests compared cells collected at 0 h to cells collected at the other time points (3, 6, 12, and 24 h). *, *p* < 0.05; **, *p* < 0.01; ***, *p* < 0.001; ****, *p* < 0.0001. Error bars, S.D.

Previously, we showed that HIV-1 gene expression is characterized by stochastic variability which results in paused polymerase and intermittent transition from latent to active state that allows the synthesis of mostly short non-coding RNAs (i.e., TAR) [19,20,36,37,38]. Paused RNA polymerase II allows synthesis of at least four distinct species of RNA that form a specific set of secondary structures [16,28,39]. Here, we isolated RNA from both intracellular and extracellular environments and RT-qPCRed for the presence of TAR, TAR-*gag* and *env* genes. The data in Figure 2A show presence of all three transcripts at 3 h, but a gradual and significant increase of all three populations over 24 h. As expected, the TAR levels were increased the most since this shows basal transcription prior to synthesis of full-length genomic RNA. The copy numbers ranged from 5 × 10^5^–6 × 10^6^ TAR RNA/mL. On the other hand, the levels of intracellular RNA increased more dramatically over time for all three populations (Figure 2B). The increase of TAR was more apparent over time (3 h vs. 24 h) which might be an indication of either increased half-life of accumulated TAR, inefficient activated polymerase II transcription, or regulation (soaking up) of Tat-activated transcription at time of viral assembly and release. Collectively, these data indicate that, similarly to viral protein increase in the extracellular environment, there was a time-dependent accumulation of all three classes of RNA over time in the induced released cells.

### 3.3. Functional HIV-1 Rescue from Released Cells

**Figure 3:**
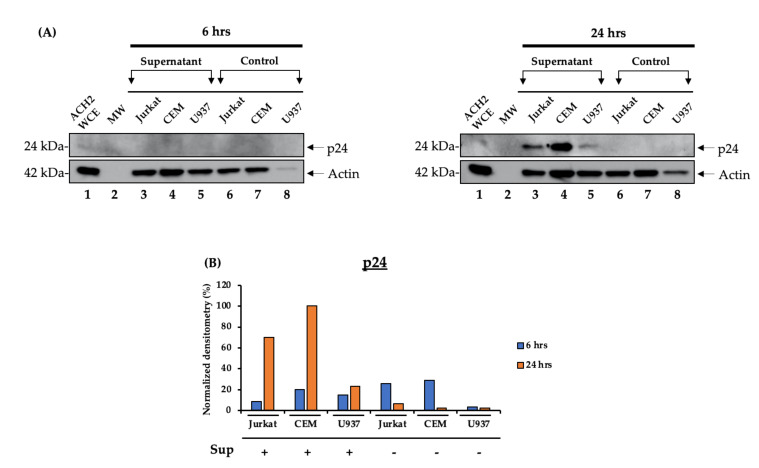
HIV-1-infected cells produce EVs containing viral proteins and RNA prior to virion release. J1.1 cells (5 × 10^7^ cells/mL) were cultured in 1% fetal bovine serum (FBS) media and treated with cART for 72 h, washed and placed in EV-depleted, 20% FBS complete media containing PHA/IL-2 to induce HIV-1 gene expression. The 6- and 24-h samples were harvested to perform an infectivity assay. (**A**) The culture supernatants were harvested at 6 and 24 h. The J1.1 (HIV-1-infected T cells) samples from 6 and 24 h were used to treat naïve CEM, Jurkat, and U937 cells. A total of 10^6^ naïve cells were resuspended in 400 µL supernatant and incubated for two days. Afterwards, 600 µL fresh complete media was added and the cells were incubated for two days. The cells were then harvested and pelleted for Western blot analysis. (**B**) Densitometry count was used to measure the level of p24 expression. WCE: whole cell extract; MW: molecular weight.

The data in Figure 1 indicate that viral proteins such as p24 were released starting at 6 h and continuing for up to 24 h. Here, we asked whether the p24+ supernatant contained functional viral particles by using infection of three cell types. We incubated early (6 h) and late (24 h) samples with two uninfected T cell lines (Jurkat and CEM) and one myeloid cell line (U937) for 48 h at 37 °C. Following potential infection from the 6- and 24-h samples, the cells were pelleted, washed, lysed, and ran on a 4–20% SDS-PAGE followed by Western blotting with an anti-p24 antibody. The results in Figure 3 show that only the 24-h samples but not the 6-h samples contained potential viral particles that, upon entry into susceptible recipient cells, were able to produce viral proteins. All three cell types exhibited p24 positivity, although it was higher in T cells than in myeloid U937 cells. Collectively, these data indicate that although 6-h samples may be positive for viral proteins or RNA, they are not generally infectious, whereas 24-h samples contain both functional viral particles and EVs.

### 3.4. Evaluation of Viral and EV Release from Other Retrovirally Infected Cells (HTLV-1)

**Figure 4:**
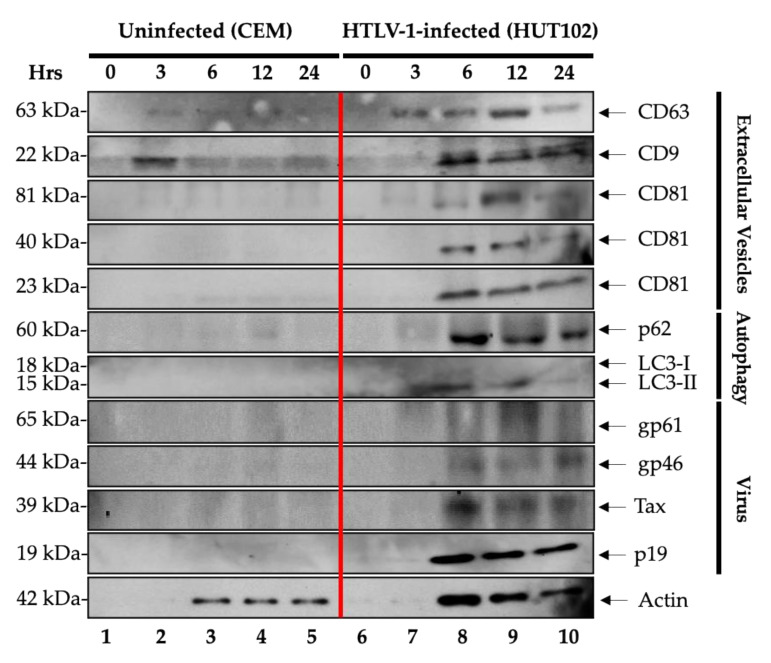
EV and virus production in HTLV-1-infected cells plateaus 6 h post-release. CEM and HTLV-1 cells (5 × 10^7^ cells/mL) were synchronized at the G_0_ stage of the cell cycle following incubation in low serum (1% fetal bovine serum (FBS)) media for 72 h and subsequently washed and incubated in EV-depleted 20% FBS media. Prior to harvesting, the cells were treated with exogenous IL-2 and PHA to increase viral transcription. Viral particles and EVs were isolated from cell supernatants (CEM (lanes 1–5) and J1.1 (lanes 6–10)) at 0, 3, 6, 12, and 24 h using Nanotraps (NT80/82/86) with overnight incubation at 4 °C. The samples were then subjected to Western blotting with primary antibodies specific for exosomal markers (CD63, CD9, and CD81), autophagy markers (p62, LC3-I and LC3-II), HTLV-1 viral proteins (gp46/61, Tax, and p19), and actin as a control.

HTLV-1 is a human retrovirus that integrates into the host genome and, similarly to HIV-1, expresses transcripts from its viral long terminal repeat (LTR) promoter. We asked whether the EV and virus release may be similar in other human retrovirally infected cells. Similar to HIV-1-infected cells, we used HTLV-1-infected HUT102 cells, which contained an integrated copy of genomic virus [40] and could be induced to make virion progeny particles. Again, we blocked these cells at G_0_ with serum starvation and released samples in the presence of complete media containing 20% serum. We also added exogenous IL-2 and PHA, collected the supernatants, and enriched EVs and virions using Nanotraps NT80/82/86. The samples were then Western blotted for presence of exosomes, autophagy, and viral markers. The data in Figure 4 demonstrate that CD63, CD9 as well as all forms of CD81 were induced at 6 h post-release from the HTLV-1-infected cells. Similar to the HIV-1-infected cells, both p62 and LC3-I/II also increased after 6 h from the HUT102 cells. Importantly, viral protein markers such as gp61/46, Tax, and p19 were also all present in 6-h samples up to 24 h. However, the levels of viral proteins did not significantly increase when compared to the HIV-1-infected cells released, indicating that the mode of EV/virus release from HTLV-1-infected cells may be distinctly different from HIV-1-infected cells. This would be consistent with the idea that HIV-1 free particles are more infectious than HTLV-1 ones where the majority of the viral transmission is through cell-to-cell contact [14]. Finally, previously, we showed that G_0_-blocked and released cells enter the G_1_ phase of cell cycle as evident by phosphorylation of retinoblastoma proteins [41,42]. Collectively, these data indicate that, similarly to HIV-1-infected released cells, HTLV-1-infected cells also release their EV and viral protein cargoes at 6 h after G_0_ release.

### 3.5. Detection of HTLV-1 RNA in Both Intracellular and Extracellular Environments

**Figure 5:**
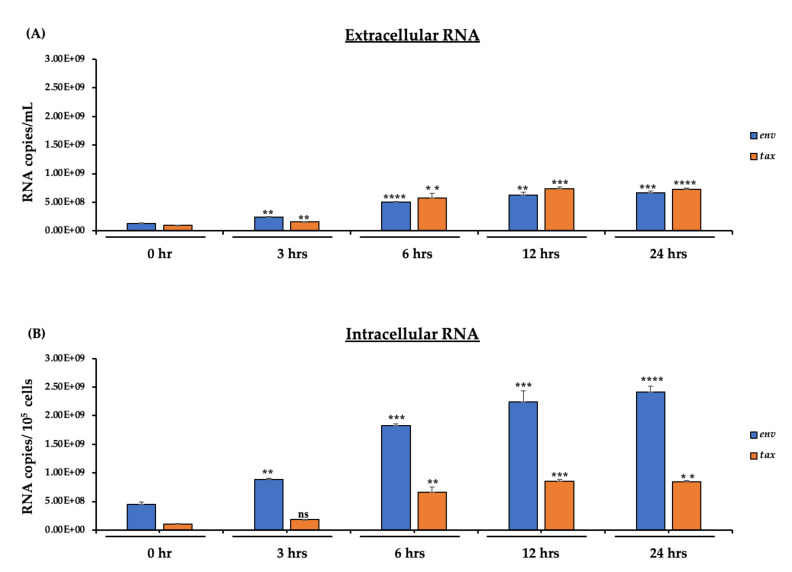
Levels of viral RNA content of secreted EVs and virions from the HTLV-1-infected cells. The HTLV-1-infected cells (5 × 10^7^ cells/mL) were cultured in 1% fetal bovine serum (FBS) media for 72 h and then washed and further incubated in EV-depleted, 20% FBS complete media to induce HTLV-1 reactivation. Prior to harvesting, the cells were treated with exogenous IL-2 and PHA to increase viral transcription. (**A**) RT-qPCR analysis for the presence of HTLV-1 *tax* and *env* RNA was performed on nanotrapped pellets (extracellular; NT80/82/86) isolated from the HTLV-1-infected cell supernatants at different timepoints (0, 3, 6, 12, and 24 h). (**B**) RNA was isolated from HTLV-1 cells (intracellular) at 0, 3, 6, 12, and 24 h for analysis by RT-qPCR of *tax* and *env*. RT-qPCRs were performed in technical triplicates. Student’s t-tests compared cells collected at 0 h to those collected at the other time points (3, 6, 12, and 24 h). **, *p* < 0.01; ***, *p* < 0.001; ****, *p* < 0.0001. Error bars, S.D.

We previously observed that much like HIV-1-infected cells, the HTLV-1 p19 protein is released starting at 6 h. Here, we asked whether these data could correlate to RNA levels from HTLV-1-infected induced cells using RT-qPCR against both *env* and *tax* regions. The data in Figure 5A show that both RNAs were released in almost equal amounts starting at 3 h and increased up to 24 h. One notable difference with HTLV-1 transcripts was that there was an almost equal increase of both *tax* and *env* RNAs over time, indicating that HIV-1 and HTLV-1 EVs feature different mechanisms of release. The data in Figure 5B indicate that there was considerably more *env* RNA compared to *tax*. Furthermore, there was a clear increase of both *env* and *tax* RNA over the 24-h period, indicating active transcription from the viral LTR promoter post-induction. Collectively, these data imply that HTLV-1 RNA synthesis is induced post-release/induction and RNA can be found in the extracellular environment.

### 3.6. Virus Rescue Assay Using Various Susceptible Cells

**Figure 6:**
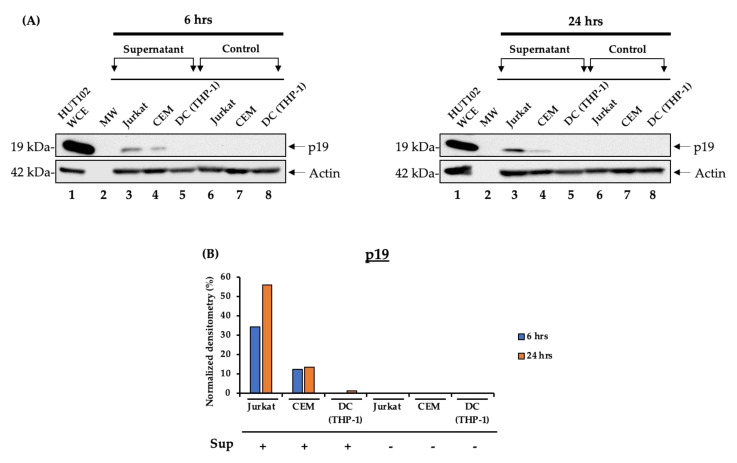
Infectivity assay of condition medium samples from HTLV-1-infected cells. HUT102 cells (5 × 10^7^ cells/mL) were cultured in 1% fetal bovine serum (FBS) media for 72 h, washed and placed in EV-depleted, 20% FBS complete media containing PHA/IL-2 to induce HTLV-1 gene expression. The 6- and 24-h samples were harvested to perform an infectivity assay. (**A**) HUT102 supernatants harvested at 6 and 24 h were used to treat naïve CEM, Jurkat, and THP-1 derived dendritic cells. A total of 10^6^ naïve cells were resuspended in 400 µL supernatant and incubated for two days. Afterwards, the supernatant was removed and 600 µL fresh complete media was added. The cells were incubated for two more days and pelleted for Western blot analysis for HTLV-1 p19 protein. (**B**) Densitometry count was used to measure the level of p19 expression.

Previous data indicated that HTLV-1 RNA could be found in the extracellular environment post-induction, and, similarly to HIV-1 rescue experiments in Figure 3, we added these supernatants to susceptible cells including uninfected T cells (Jurkat and CEM) and dendritic cells (THP-1-induced DCs). The cells were incubated for 48 h, followed by wash and incubation with complete media for an additional 48 h. The cells were pelleted, lysed, separated on a 4–20% SDS-PAGE and Western blotted for the presence of p19 and actin. The data in Figure 6A indicate that samples from the 6-h supernatants as well as the 24-h samples exhibited replication in the Jurkat cells and very little in the CEM cells, and no replication in DCs. However, there was increased replication from the 24-h samples, which is consistent with the notion that more viral particles may be released at later times. The data in Figure 6B quantify the p19 protein levels from recipient cells. We finally asked whether the particle (EV and virions alike) concentration changes overtime. Nanoparticle tracking analysis (NTA; ZetaView) was utilized to quantify EVs released from the uninfected (CEM), HIV-1-infected (J1.1) and HTLV-1-infected (HUT102) cells (Figure S1). All three cell lines exhibited increased concentration of particles over time that peaked at 24 h.

### 3.7. Lack of Cell Death Following Release with Complete Media

**Figure 7:**
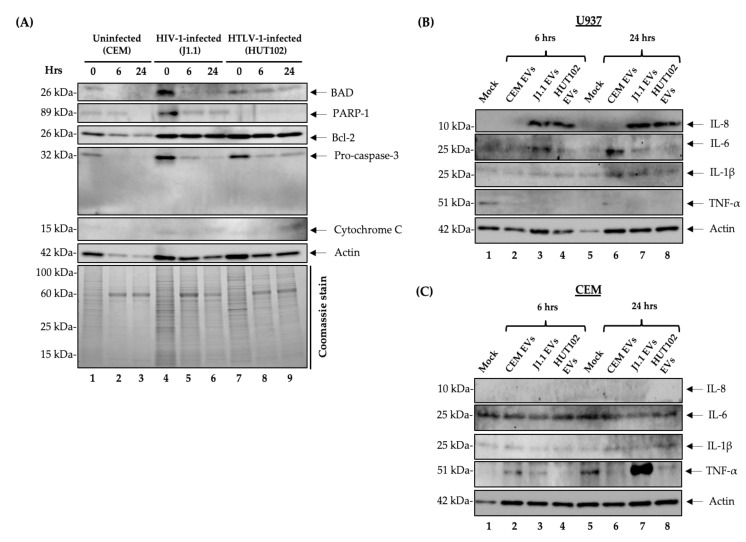
Expression of apoptotic and cytokine markers in the treated recipient cells. (**A**) CEM, J1.1, and HUT102 cells (5 × 10^7^ cells/mL) were synchronized at G_0_ by serum starvation. The J1.1 cells were treated with cART to induce latency. Three days later, all three cell lines were cultured with complete media containing 20% EV-depleted serum and PHA/IL-2 to increase viral transcription. The cell pellets were collected at 0, 6, and 24 h post-induction. Cell lysates were analyzed by Western blotting for apoptotic markers (BAD, PARP-1, Bcl-2, pro-caspase-3, and cytochrome C) and actin as a control. An image of the Coomassie stained gel is shown to reflect the protein samples loaded. (**B**) One hundred thousand U937 cells and (**C**) CEM cells (recipient cells) were plated and treated with total EVs derived from the CEM, HIV-1-, and HTLV-1-infected cells released at 6 and 24 h. The second treatment with EVs was performed 24 h later. The recipient cells were incubated with EVs for 48 h. The total ratio of cells to EVs was 1:10^4^. The supernatants were collected and EVs were nanotrapped (NT80/82) overnight. Western blots were performed for IL-8, IL-6, IL-1β, TNF-α, and actin.

To assess cell viability, we decided to test the cells for the presence of apoptotic markers before and after release (i.e., BAD, PARP-1, Bcl-2, pro-caspase-3, and cytochrome C) (Figure 7A). Following induction, the CEM, J1.1, and HUT102 cells were harvested at 0, 6, and 24 h, pelleted, and lysed. Next, we performed Western blot analysis and probed the samples for apoptotic markers. In the HIV-1-infected cells, we observed that BAD, PARP-1, and pro-caspase-3 were ubiquitously expressed at 0 h (lane 4) but decreased at 6 and 24 h. In the HTLV-1-infected cells, BAD and PARP-1 did not exhibit any significant change in expression levels over time. Pro-caspase-3 expression was highest at 0 h (lane 7) but dramatically decreased at 6 and 24 h. The absence of active caspase-3 in the cells tested in Figure 7A suggests lack of cell death. Bcl-2 exhibited no differential expression over time in either HIV-1- or HTLV-1-infected cells and was abundant at 0, 6, and 24 h. Cytochrome C was mostly absent in all the samples tested. Actin exhibited an unusual expression pattern in the uninfected cells, perhaps due to actin solubility differences. As such, we stained the gel to assess protein quantity. Images of the Coomassie staining in Figure 7A reveal that the amounts of protein loaded were consistent between each sample, especially notably post-release. Collectively, these data suggest that cells post-release are alive and show reduced markers of apoptosis.

### 3.8. HIV-1 and HTLV-1 EVs Promote Differential Expression of IL-8 and TNF-α in Myeloid and T Cell Lines

We next asked whether there was a difference in EV function when released at early or later stages on recipient cells. Therefore, the mixtures of total EVs collected from the uninfected (CEM), HIV-1-infected (J1.1), and HTLV-1-infected (HUT102) cells at 6 and 24 h post-release were added to the recipient uninfected myeloid (U937) (Figure 7B) and T (CEM) (Figure 7C) cell lines at a cell to EV ratio of 1:10^4^. Previously, we observed this cell/EV ratio to be optimal for functional effects (data not shown). The samples were incubated for 48 h and the supernatants were collected and enriched with a NT80/82 nanoparticle cocktail. Next, we performed Western blot analysis on the supernatants of each cell line and probed for the presence of IL-8, IL-6, IL-1β, TNF-α, and actin. We observed an abundance of IL-8 in myeloid cells treated with the J1.1 and HUT102 EVs collected at 6 h (Figure 7B, lanes 3 and 4). Higher expression was present in myeloid cells treated with the J1.1 and HUT102 EVs collected at 24 h (Figure 7B, lanes 7 and 8). However, IL-8 was completely absent in the EV-treated CEM cells (Figure 7C). The U937 cells expressed IL-6 when treated with the J1.1 EVs collected at 6 h (Figure 7B, lane 3) and the CEM EVs collected at 24 h (Figure 7B, lane 6). Interestingly, T cells consistently expressed IL-6 regardless of the EV treatment type. Neither U937 nor CEM cell lines exhibited differential expression of IL-1β upon treatment with EVs. Finally, TNF-α expression was observed only in the CEM cells treated with the CEM EVs collected at 6 h (Figure 7C, lane 2) and the J1.1 EVs collected at 24 h (Figure 7C, lane 7).

### 3.9. EV Contents from Infected Primary Cells

**Figure 8:**
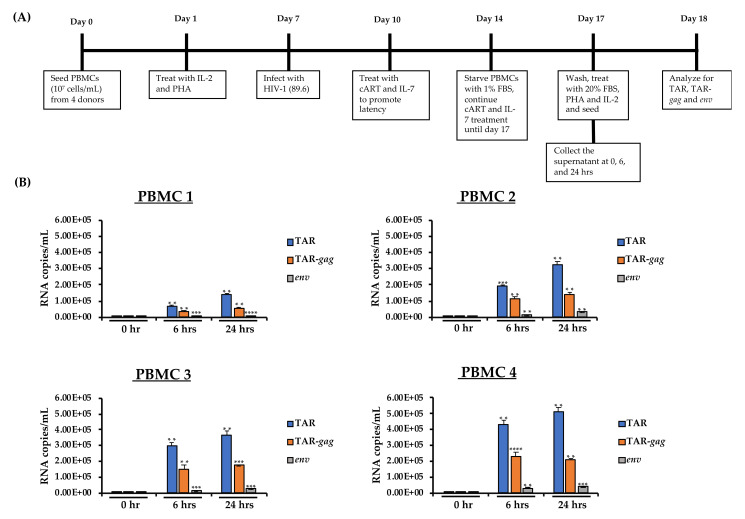
Production of extracellular viral RNAs from HIV-1-infected primary cells post-release. (**A**) The experimental design used for primary cell infection and EV collection. PBMCs from four independent donors were treated with PHA and IL-2 and allowed to grow for seven days in culture. The PBMCs were then infected with HIV-1 89.6 (MOI: 10) and treated with cART and IL-7 to promote latency. The cells were then synchronized in G_0_ by serum starvation for three days and induced with EV-depleted, 20% FBS complete media containing PHA/IL-2. The samples were harvested at 0, 6, and 24 h post-induction and enriched for virus and EVs using NT80/82/86 nanoparticles. (**B**) Total RNA from nanotrapped pellets was isolated. Using 3′-end primers specific to TAR, TAR-*gag* and *env* regions, cDNA was produced. RNA levels were assessed by RT-qPCR with TAR-specific primers. Student’s t-tests compared cells collected at 0 h to cells collected at the other time points (6 and 24 h). **, *p* < 0.01; ***, *p* < 0.001; ****, *p* < 0.0001. Error bars, S.D.

We cultured PBMCs from four different donors for seven days with the addition of PHA/IL-2 every other day (Figure 8A). The PBMCs were then infected with 89.6 dual-tropic HIV-1 (MOI: 10). PHA/IL-2 treatment continued for another three days. The PBMCs were then forced into latency using cART and IL-7 treatment over the course of three days. On day 6 post-infection, the PBMCs were synchronized at G_0_ via serum starvation for three days, while cART/IL-7 treatment continued. Next, the cells were plated in 20% complete media and induced with PHA/IL-2 to reverse latency and quiescence. Supernatant samples were harvested at 0, 6, and 24 h post-induction. Nanoparticles NT80/82/86 were used to enrich samples for virions and EVs for RT-qPCR. RNA was extracted and processed for RT-qPCR as described previously [30]. The data in Figure 8B indicate that TAR was detected at 6 and 24 h, peaking at 24 h in all four PBMCs. Interestingly, TAR-*gag* expression was detected both at 6 and 24 h; however, its expression did not differ significantly in the 6- and 24-h samples. Genomic RNA was found in the 6- and 24-h samples and showed increase over time. Overall, RT-qPCR data from condition medium samples from the PBMCs showed gradual increase in concentration of HIV-1 RNAs over time peaking at 24 h, except for TAR-*gag* that remained stable at 6 and 24 h.

## 4. Discussion

Extracellular vesicles, including exosomes from virally infected cells, carry viral proteins and RNAs, are taken up by naïve cells and alter their physiology. Our previous works revealed a role of EVs in HIV-1 pathogenesis where exosomes from infected cells transferred viral RNAs and proteins to neighboring cells, leading to either activation or deleterious consequences [19,31]. Furthermore, EVs were found to prime the environment to support viral spread. As such, it has become critical to understand the dynamics of EV and virus release in a timely manner. Here, we addressed this issue by studying the content and population of particles released by virally infected vs. uninfected cells.

Our data showed that there was an increase in production of tetraspanin proteins, namely CD63, CD81, and CD9 from HIV-1-infected cells (Figure 1). This differential expression of exosomal markers in virally infected cells supports the findings that HIV-1 recruits tetraspanins to facilitate its life cycle [43,44]. Interestingly, we observed a couple of similarities in the data presented in Figure 1 and Figure 4 regarding tetraspanin expression in both HIV-1 and HTLV-1 EVs compared to their uninfected counterparts. CD63 increased starting at 3 h (Figure 1) while CD81 expression was observed to be higher in infected EVs than in uninfected EVs (Figure 1 and Figure 4). On the other hand, CD9 initially increased then decreased after 12 h in infected EVs. These changes in EV marker expression over time could potentially be due to the increase of EV release at different timepoints (Figure S1). Furthermore, this differential tetraspanin expression could also be attributed to the concept that cells produce different EV populations that are heterogenous in their protein markers [18,30] and that tetraspanins such as CD9 could potentially enhance viral spread and EV biogenesis [45]. Moreover, viral capsid protein p24 was found to interact with CD81 and colocalize in tetraspanin-enriched microdomains composed of CD63, CD9, and CD81, which is crucial for virion assembly [46,47]. Autophagy-related proteins, namely p62 and LC3-I/II, are indicative of autophagosome production and release from infected cells. The data in Figure 1 show that p62 and LC3-I/II were released in higher quantities from the infected cells, consistent with the findings that HIV-1-infected cells enable abundant gene expression, which may be regulated by the autophagy pathway. For instance, cells that contain abundant levels of viral RNA or proteins would either have to degrade unwanted viral RNA or proteins or alternatively sequester, package and transport them to the extracellular environment through secretory autophagy. The latter may be more physiological as viral proteins such as Env, Tat, Nef, and Vpr, among others, have been shown to both modulate autophagy, thereby inhibiting degradation of viral components, or exploit the autophagy machinery, resulting in its upregulation through an Env-dependent mechanism [35]. Interestingly, the results from Figure 1 demonstrate the upregulation of gp120, Nef, p24, and Pr55 over time, with the highest expression observed at 24 h. This could indicate that markers of EVs are present prior to viral protein markers, supporting the hypothesis that EVs are released prior to virion release. Overall, our data suggest that HIV-1 infection dramatically changes the host protein expression patterns where tetraspanin family members and autophagy-related proteins are upregulated in HIV-1-infected cells.

Our previous works showed that HIV-1 transcription is intermittent and characterized by multiple premature terminations, resulting in the production of multiple short and long non-coding RNA species that are able to form secondary structures [20,39]. In this study, we evaluated viral RNA production in both the extracellular and intracellular environments by assessing the RNA levels of TAR, TAR-*gag,* and genomic RNA from the 0-, 3-, 6-, 12- and 24-h samples using RT-qPCR. As expected, the results in Figure 2A reveal that among all three extracellular RNA populations, the TAR levels (indicative of basal transcription) were increased the most. Long non-coding TAR-*gag* RNA was the second most abundant transcript, followed by full genomic *env* RNA. The production of TAR was the most effective over time, which might imply either a decreased decay rate of accumulated TAR, inefficient activated RNA polymerase II transcription or regulation of Tat-activated transcription at the time of viral assembly and release. Similar results were observed in Figure 2B where intracellular TAR, TAR-*gag,* and genomic RNA were assayed. Although particle populations from the extracellular environment released at 3, 6, and 12 h contained full genomic HIV-1 RNA, it may not necessarily indicate the presence of fully infectious virions as various RNA populations have been found to be packaged in EVs of different sizes through LC3 conjugation machinery with recruitment of RNA-binding proteins [48]. Additionally, Pr55 has been shown to be responsible for packaging and transporting genomic HIV-1 RNA into virions [49]. As such, genomic RNA found in EVs was most likely transported there by Pr55 [30]. Together, these data demonstrate that there was a time-dependent accumulation of all three classes of RNA over time in induced released cells.

We observed that the 24-h samples contained a population of particles that are able to induce viral protein production in naïve cells as compared to the 6-h samples (Figure 3A,B). Interestingly, the resulting p24 was expressed in higher quantities in T cells than in myeloid cells as demonstrated in Figure 3B. This supports the findings that monocytes and macrophages are more resistant to productive HIV-1 infection due to differential expression of multiple host restriction factors [50]. The virus rescue data suggest that although the 6-h samples tested positive for viral proteins and genomic RNA (Figure 1 and Figure 2A), they may not have been infectious. On the other hand, the 24-h samples were shown to contain functional virions that are capable of productive infection of naïve cells. The infectivity of the 24-h samples but not of the 6-h samples could be augmented by the presence of EVs. This is in line with data from Figure 1 (lane 10) that demonstrate that the concentration of EVs peaks at 24 h. Collectively, these results imply that virions released at 24 h are indeed infectious, and the presence of EVs may contribute to their infectivity, since EVs from virally infected cells have been found to contain viral proteins and RNA that can increase the pool of surrounding cells susceptible to infection [14,18,19,20,32,51,52].

As shown in Figure 4, the HTLV-1-infected cells were confirmed to secrete EVs that contain Tax and gp46/61 proteins. This confirms the previous findings of EV-associated Tax and gp46/61 in cell lines, PBMCs, and CSF from HAM/TSP patients [14,32,53]. Similar to the HIV-1-infected cells, tetraspanin proteins were differentially produced by the HTLV-1-infected cells over time in comparison to the uninfected cells. EV markers including CD63 were observed at an earlier timepoint (3 h) in comparison to viral proteins such as Tax and p19 (Figure 4). HTLV-1 is likely to recruit tetraspanin proteins to assist virion assembly. The HTLV-1 Gag protein was found to interact with the intracellular loop of CD81, which mediates the association of HTLV-1 with tetraspanin-enriched microdomains that contain other tetraspanins such as CD9 and CD63 [54]. In addition, our data revealed autophagy-related proteins, namely LC3-I/II and p62, to be differentially expressed in HTLV-1-infected cells (Figure 4). Previously, the Tax protein was found to recruit numerous autophagic proteins and upregulate LC3+ autophagosome assembly in the proximity of lipid raft microdomains, resulting in the deregulation of autophagy and enhanced viral spread [55]. This could potentially explain the increased production of LC3-I/II we observed in the extracellular environment. Taken together, these data demonstrate that, similar to HIV-1-infected cells, HTLV-1-infected cells release EVs prior to viral proteins and upregulate autophagic markers, which may potentially contribute to cell transformation.

The data in Figure 5A,B show that the concentration of HTLV-1 RNA in both the intracellular and extracellular environments began to increase at 6 h; however, it stayed the same up to 24 h. This was in contrast to the HIV-1 expression in Figure 2A,B where viral RNA dramatically increased by the 24-h timepoint. This indicates that there may be different mechanisms of EV release in retroviruses (i.e., between HIV-1 and HTLV-1). Furthermore, there was considerably more *env* RNA compared to *tax* RNA in both the intracellular and extracellular environments. There was a clear increase of both *env* and *tax* RNA over the 24-h period, indicating active transcription from viral LTR promoter post-induction. This could potentially be attributed to Tax’s oncogenic role in viral transcription and spread where it has been found that Tax contributes to the abnormal proliferation of HTLV-1-infected cells and T cell transformation [56,57]. Lastly, we observed that the HTLV-1-infected cells contained higher intracellular *env* RNA compared to secreted *env* RNA (EV-bound) at each timepoint (Figure 5A,B). This is consistent with observations that HTLV-1 primarily exists intracellularly, and that infectious virion release occurs sporadically in infected cells [14,18,56,58]. Collectively, these data indicate that HTLV-1 RNA synthesis is increased post-release/induction and that viral RNA can be found in the extracellular environment.

Interestingly, supernatants from both the 6- and 24-h samples showed the induction of p19 in naïve susceptible T cell lines (Jurkat and CEM), with notably higher p19 expression upon the treatment by the 24-h sample (Figure 6). This indicates that more virions are released and accumulated at a later time after induction. The THP-1-derived dendritic cells did not show susceptibility to both the 6- and 24-h samples, which might have been caused by various pre- and post-entry restriction factors related to DC maturation processes [14]. Finally, it may be worth noting that, unlike the HIV-1 promoter, HTLV-1 gene expression is difficult to completely suppress as the promoter is consistently active even under serum starvation conditions [59,60]. This could potentially explain our observations of viral rescue in both the 6- and 24-h samples (Figure 6A,B). Altogether, our data suggest that viral rescue can be observed in susceptible T cells when using p19 expression.

As indicated previously, intracellular protein expression occurs differentially and correlates with time post-induction. Several apoptotic markers are expressed differentially in virally infected cells over time post-induction, with the highest expression at the end of the block (Figure 7A). This pattern of apoptotic protein expression is consistent with the findings that many cells at the end of the block are subjects to serum starvation-induced apoptosis [61,62]. The decrease of apoptotic markers, namely BAD, PARP-1 and pro-caspase-3, is likely caused by the release of cells from starvation which occurs when cells are placed into serum-rich media with PHA and IL-2 [63,64]. Pro-inflammatory cytokines have been implicated in the pathogenesis of HIV-1 and progression to AIDS due to compromised cell-mediated immune response [65,66]. Therefore, we tested EVs from infected cells released at different timepoints on their ability to modulate proinflammatory cytokine production. The data in Figure 7B show IL-8 was upregulated in myeloid cells (U937) upon treatment with EVs from the HIV-1- and HTLV-1-infected cells alike. In the case of HIV-1, this could stem from the function of TAR as it was previously found that EV-associated HIV TAR RNA is responsible for the upregulation of IL-8 and TNF-α production in recipient cells [20]. For instance, EVs from HIV-1-infected cells have been demonstrated to contain HIV-1 TAR, which binds to PKR and TLR-3, leading to increased cytokine production and a compromised innate immune response [20]. The increased expression of IL-8 in the U937 cells treated with HTLV-1 EVs (6 and 24 h) could be caused by EV-associated viral RNA (yet undefined) or the HTLV-1 Tax protein that has been found to induce IL-8 expression [67,68]. Lastly, trimeric membrane-associated TNF-α was found in the T cells (CEM) treated with EVs from the HIV-1-infected cells released at 24 h (Figure 7C, lane 7), which is suggestive of activation of the TNF pathways, possibly due to infection. However, it is also possible that the upregulation of TNF-α may be caused by single viral product(s) differentially packaged within EVs.

The investigation of RNA contents of condition medium samples from the HIV-1-infected primary cells from four independent donors revealed a gradual increase in concentration of all three HIV-1 RNA populations over time (Figure 8). However, TAR-*gag* RNA showed the lowest increase between 6 and 24 h in comparison to TAR and genomic RNAs. This could be attributed to the physiological features of primary cells in comparison to immortalized cell lines (i.e., J1.1). Alternatively, the TAR-*gag* RNA may be differentially regulated either inside or outside of cells, as they are known to be a landing pad for other proteins that could regulate epigenetics and gene expression [39].

**Figure 9:**
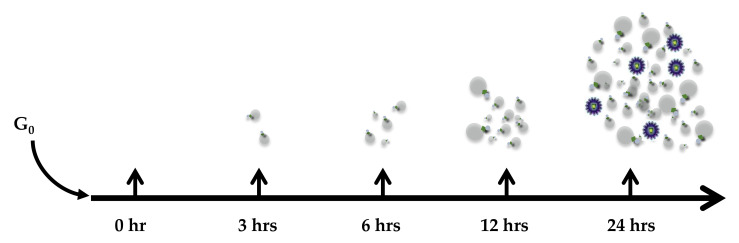
Proposed model for virion and EV release dynamics. Small EVs are the first to be released by infected cells, and viral proteins and RNAs are packaged into these EVs. Although the mechanism of this release and lack of intracellular digestion is not clear, it is speculated that viral RNAs or proteins that are made first (i.e., TAR, or products of doubly spliced messengers including Nef) potentially aid in autophagy inhibition of the host cell or promote EV release from infected cells. The viral products in these EVs are not infectious. However, at a later time, the number of released EVs increase along with the few viral particles that are infectious. The diagram also assumes that the majority of particles at 24 h are EVs containing viral products (RNA or proteins) and not fully infectious virions.

In summary, we found that virally infected cells release EVs containing viral proteins and RNA transcripts prior to releasing virions when entering normal cell cycle (Figure 9). As most lymphocytes are dormant in the G_0_ phase, including the ones infected with HIV-1, it is important to address the question of whether EVs carrying viral proteins and RNA transcripts “prepare” recipient cells for the next cycle of viral infection and assist in viral spread. Overall, our results are consistent with the hypothesis that EVs from HIV-1- and HTLV-1-infected cells are released prior to virions. These early EVs contain viral proteins and RNAs that can activate the cell cycle and render naïve recipient T cells and monocytes more vulnerable to subsequent viral infection. Altogether, these data could allow further understanding of the effects of EVs on naïve recipient cells [18,69] and contribute to knowledge about the effects of EVs in enhancing susceptibility to infection. More importantly, these findings show the importance of EVs during viral infection, since they are secreted before the virions and contain several viral proteins with the potential to induce inflammation and disease. Future experiments will define which precise times show specific release of viral RNA or proteins that may correlate with the blocking of the autophagy pathway and whether the 24-h samples also have large EVs that contain viral particles for differentiated entry into cells.

## Data Availability

The data presented in this study are available on reasonable request from the corresponding author.

## Supplementary Materials

The following are available online at https://www.mdpi.com/article/10.3390/cells10040781/s1, Figure S1: EV Concentration Increases Over Time.

## Abbreviations

EV	extracellular vesicle
PHA	phytohemagglutinin
cART	combination antiretroviral therapy
TAR	transactivation response
ESCRT	endosomal sorting complex required for transport
HAND	HIV-1-associated neurocognitive disorder
RT-qPCR	real-time quantitative polymerase chain reaction
HAM/TSP	HTLV-1-associated myelopathy/tropical spastic paraparesis
NTA	nanoparticle tracking analysis
PBMC	peripheral blood mononuclear cell
