# Supplementary material for: Extracellular Vesicles from Infected Cells Are Released Prior to Virion Release
Source: Cells. 2021 Apr 1;10(4):781. doi: 10.3390/cells10040781 (PMC8066806; doi:10.3390/cells10040781)
Supplement: Supplementary file 1 [file cells-10-00781-s001.pdf]

SUPPLEMENTARY MATERIALS

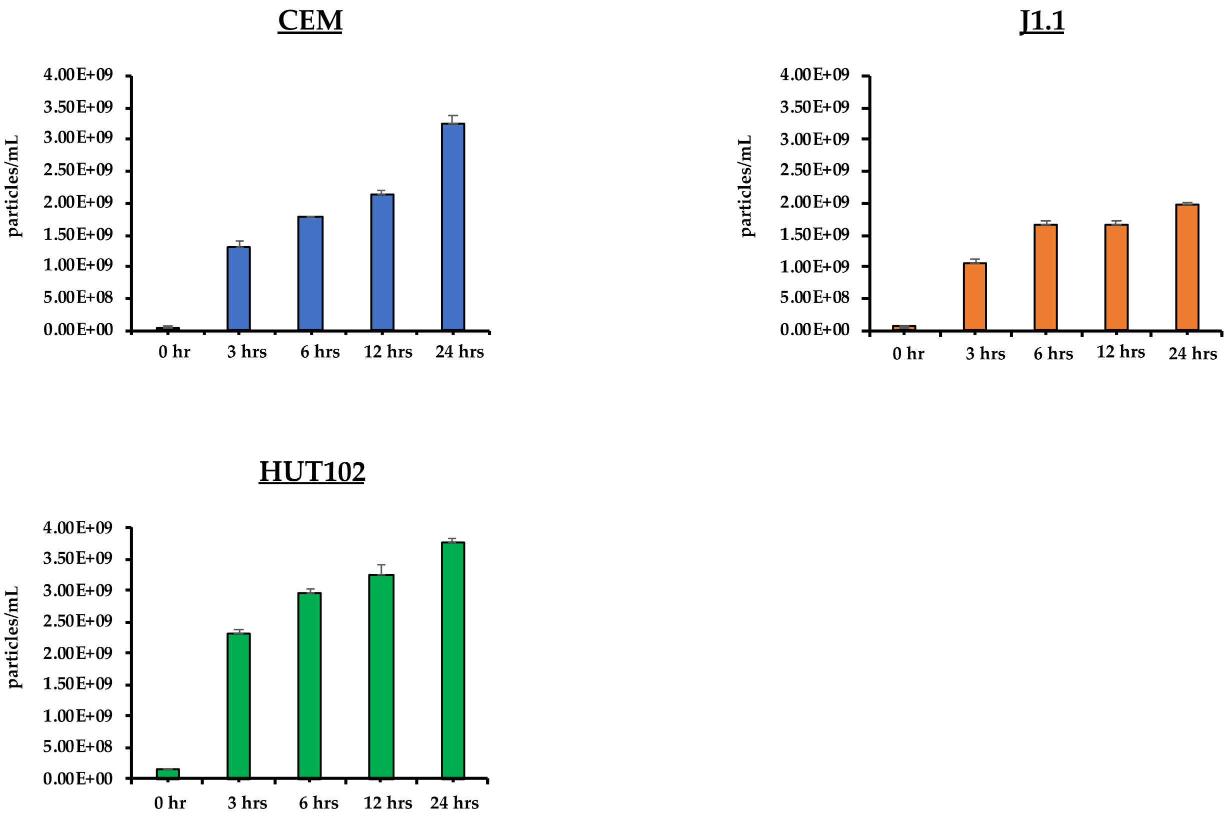

Figure S1. EV Concentration Increases Over Time. ZetaView analysis of supernatant material derived from uninfected (CEM), HIV-1-infected (J1.1), and HTLV-1-infected (HUT102) cells was performed to determine EV concentration from three technical replicates of 0-, 3-, 6-, 12-, and 24-h samples.
